# Discriminant analysis and binary logistic regression enable more accurate prediction of autism spectrum disorder than principal component analysis

**DOI:** 10.1038/s41598-022-07829-6

**Published:** 2022-03-08

**Authors:** Wail M. Hassan, Abeer Al-Dbass, Laila Al-Ayadhi, Ramesa Shafi Bhat, Afaf El-Ansary

**Affiliations:** 1 Department of Biomedical Sciences, University of Missouri-Kansas City School of Medicine, Kansas City, MO USA; 2 Biochemistry Department, College of Sciences, King Saud University, Riyadh, Saudi Arabia; 3 Department of Physiology, Faculty of Medicine, King Saud University, Riyadh, Saudi Arabia; 4 Autism Research and Treatment Center, Riyadh, Saudi Arabia; 5 Central Research Laboratory, Female Centre for Scientific and Medical Studies, King Saud University, Riyadh, Saudi Arabia

**Keywords:** Biochemistry, Neuroscience

## Abstract

Autism spectrum disorder (ASD) is a neurodevelopmental disorder characterized by impaired social interaction and restricted, repetitive behavior. Multiple studies have suggested mitochondrial dysfunction, glutamate excitotoxicity, and impaired detoxification mechanism as accepted etiological mechanisms of ASD that can be targeted for therapeutic intervention. In the current study, blood samples were collected from 40 people with autism and 40 control participants after informed consent and full approval from the Institutional Review Board of King Saud University. Sodium (Na^+^), Potassium (K^+^), lactate dehydrogenase (LDH), glutathione-s-transferase (GST), and mitochondrial respiratory chain complex I (MRC1) were measured in plasma of both groups. Predictive models were established to discriminate individuals with ASD from controls. The predictive power of these five variables, individually and in combination, was compared using the area under a ROC curve (AUC). We compared the performance of principal component analysis (PCA), discriminant analysis (DA), and binary logistic regression (BLR) as ways to combine single variables and create the predictive models. K^+^ had the highest AUC (0.801) of any single variable, followed by GST, LDH, Na^+^, and MRC1, respectively. Combining the five variables resulted in higher AUCs than those obtained using single variables across all models. Both DA and BLR were superior to PCA and comparable to each other. In our study, the combination of Na^+^, K^+^, LDH, GST, and MRC1 showed the highest promise in discriminating individuals with autism from controls. These results provide a platform that can potentially be used to verify the efficacy of our models with a larger sample size or evaluate other biomarkers.

## Introduction

The use of multivariate profiles as diagnostic biomarkers of autism spectrum disorder (ASD) is often superior to the use of individual biomarkers^1–4^. Multiple methods have been used to combine individual biomarkers into multivariate profiles. We have previously used principal component analysis (PCA) for this purpose^1^. PCA is a statistical method that aims to simplify the interpretation of high-dimensional data by displaying data points in low-dimensional space. This is accomplished by displaying the data in a new coordinate system designed to maximize the amount of variance aligned with each axis. PCA consolidates clusters of correlated variables into common dimensions, known as eigenvectors or principal components (PCs), which serve as axes in the new coordinate system. The first PC (PC1) is positioned so that it accounts for the most variance that can be explained in one dimension. PC2 is orthogonal to (i.e., uncorrelated with) PC1 and is positioned in such a way that it explains the most possible of the remaining variance. Other PCs are selected in a similar manner culminating in as many PCs as variables, ordered by the amount of explained variance^5,6^. Although the number of PCs is equal to the number of variables, most of the variation in the data are contained in the first PCs. In practice, the first two or three components account for most of the variance and can, thus, be used to graph the data in a new two- or three-dimensional coordinate system with minimal data loss. PCA transforms values across all variables using coefficients that dictate how much each variable contributes to any given principal component. This process computes a new value, known as a component score, for each data point for each PC. These scores are then used to plot the data in the new coordinate system^5^, resulting in what is commonly known as score plots. To create multivariate biomarkers, we inspected PCA score plots to identify the PC along which groups (e.g., ASD and control participants) were most distinctively separated, and used the corresponding scores as a combined biomarker^1^. The rationale is that these scores were weighted sums of the original values, with each variable contribution proportional to its correlation with the PC that accounts for most of the intergroup variance. This creates one score per subject that harbors information from all variables with proportionally greater contributions from the most discriminatory variables.

Other ways to compute multivariate biomarkers include using Z-scores. Abruzzo et al.^3^ combined variables by calculating the sum of Z-scores over all variables for each subject. We have previously compared the performance of PCA and sum of Z-scores (Eq. 1) using the *a*rea *u*nder a receiver operating characteristic (ROC) *c*urve (AUC) method and found that the use of PCA was superior^1^. Both methods rely on the variance contained in the data set without directly focusing on intergroup variance. In PCA, the orientation of PCs is aligned with maximum total variance contained in the data set. In the sum-of-Z-scores method, transformed values are reliant on dispersion around the means of variables, also, in the whole data set.

1 $$Z= \frac{x- \mu }{\sigma },$$

where *Z* is the Z-score, *x* is the observed value for any given variable, μ is the mean of the variable over all subjects, and *σ* is the standard deviation.

Discriminant analysis (DA) is conceptually similar to PCA, except that its computation is geared towards a different goal, namely the discrimination between user-defined groups. Therefore, the main difference between PCA and DA is that the former maximizes the amount of variance accounted for by each PC, while the latter maximizes group separation. In theory, DA should be superior in discriminating between groups because, unlike PCA, DA directly selects the most discriminatory eigenvectors or discriminants^6^. The mathematical basis of binary logistic regression (BLR) is conceptually distinct from both PCA and DA. Instead of defining eigenvectors, it calculates odds ratios and probabilities of falling into one of two groups (e.g., control or ASD group). The odds ratio is the probability of falling in one of the two groups divided by the probability of falling in the other; these probabilities and odds ratios are calculated for each participant. The coefficients used in the calculation of a BLR model are aimed at maximizing the model’s fit or the model’s ability to correctly classify participants into their respective groups^6^. Therefore, both DA and BLR consider the a priori knowledge of group membership, while PCA ignores such knowledge. Like PCA, DA and BLR both provide single scores for each participant that have been derived from multiple observed variables, which makes it possible to combine biomarkers using any of these techniques.

In this study, we compare the utility of DA, BLR, and PCA in creating multivariate biomarkers of ASD using first discriminant (Disc1) scores, predicted probabilities (PProb), and PC1 scores, respectively. We hypothesize that DA and BLR should show higher accuracy in distinguishing ASD and control participants compared to PCA. The goal of this study is to empirically test this hypothesis. For this purpose, we selected five analytes or variables, K^+^, Na^+^, LDH, GST, and MRC1, all of which show potential diagnostic value. These variables are directly or indirectly related to selected etiological mechanisms in ASDsuch as channelopathy, mitochondrial dysfunction, oxidative stress, and glutamate excitotoxicity^7–12^. It is well accepted that ion transport across the membrane regulates diverse and important neuronal cell functions, ranging from generation of action potential to gene expression and cell morphology. Therefore, it is not surprising that channelopathies have intense effects on ASDbrain functions^7^. Genetic analyses of individuals with ASDrevealed damaging mutations in several K^+^ channel types, which supports the notion that their down regulation may play a critical role in ASDpathogenesis^8^. It is widely accepted that K^+^, Na^+^, and H^+^ ion channels are involved in controlling mitochondrial function^13,14^, as the movement of these ions across the mitochondrial membrane is essential in establishing membrane potential and maintaining H^+^ flux. Mitochondrial channelopathies have also been causally linked to ASD pathogenesis^15^. K^+^ channels play an important role in excitotoxicity, a pathogenic mechanism that has been linked to ASD and is provoked by continuous overstimulation of glutamate receptors and oxidative neuronal damage^16–18^. Accordingly, the five selected variables of the current study have been well considered and repeatedly shown to correctly predict relevant clinical presentation of ASD across a variety of treatments and populations, thus, their use is entirely justified and appropriate.

## Results

### Initial evaluation of the analytes

**Figure 1:**
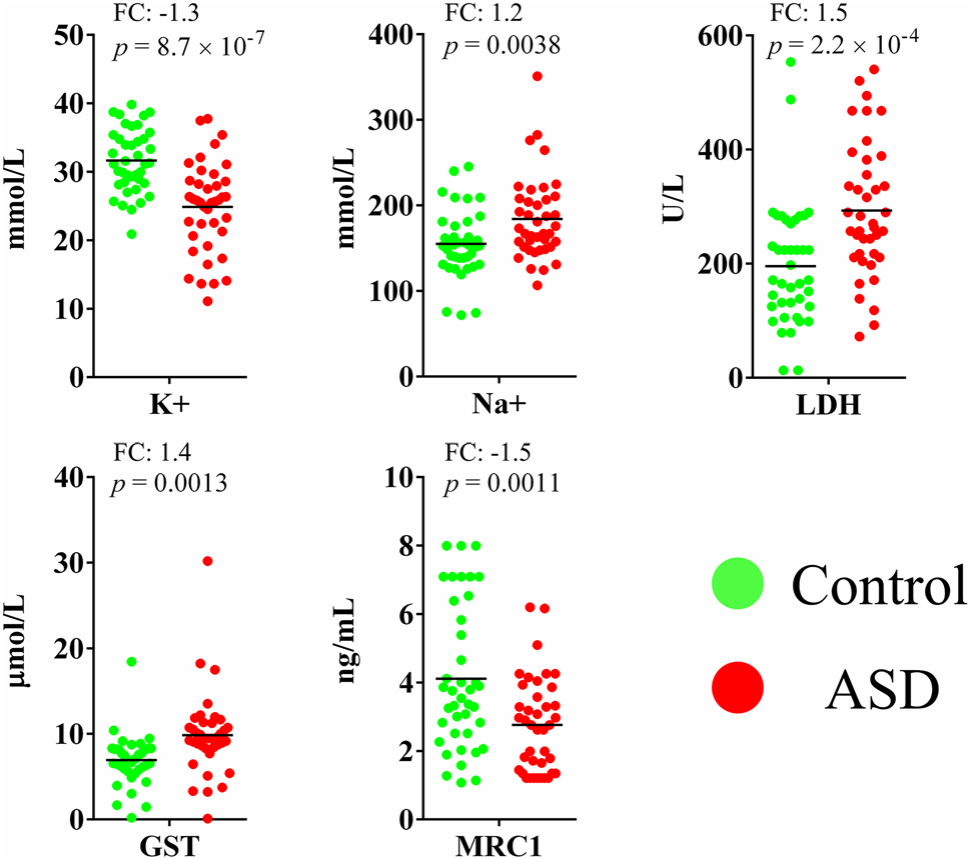
Five biomarkers differ between ASD patients (n = 40) and healthy controls (n = 40). Potassium (K), sodium (Na), lactate dehydrogenase (LDH), glutathione S-transferase (GST), and mitochondrial respiratory chain complex I (MRC1) were measured in plasma samples collected from autistic and age-matched healthy volunteers. A two-tailed student’s t-test was used to estimate statistical significance. Figure was generated in GraphPad Prism version 6 for Windows, GraphPad Software, San Diego California USA, https://www.graphpad.com.

**Figure 2:**
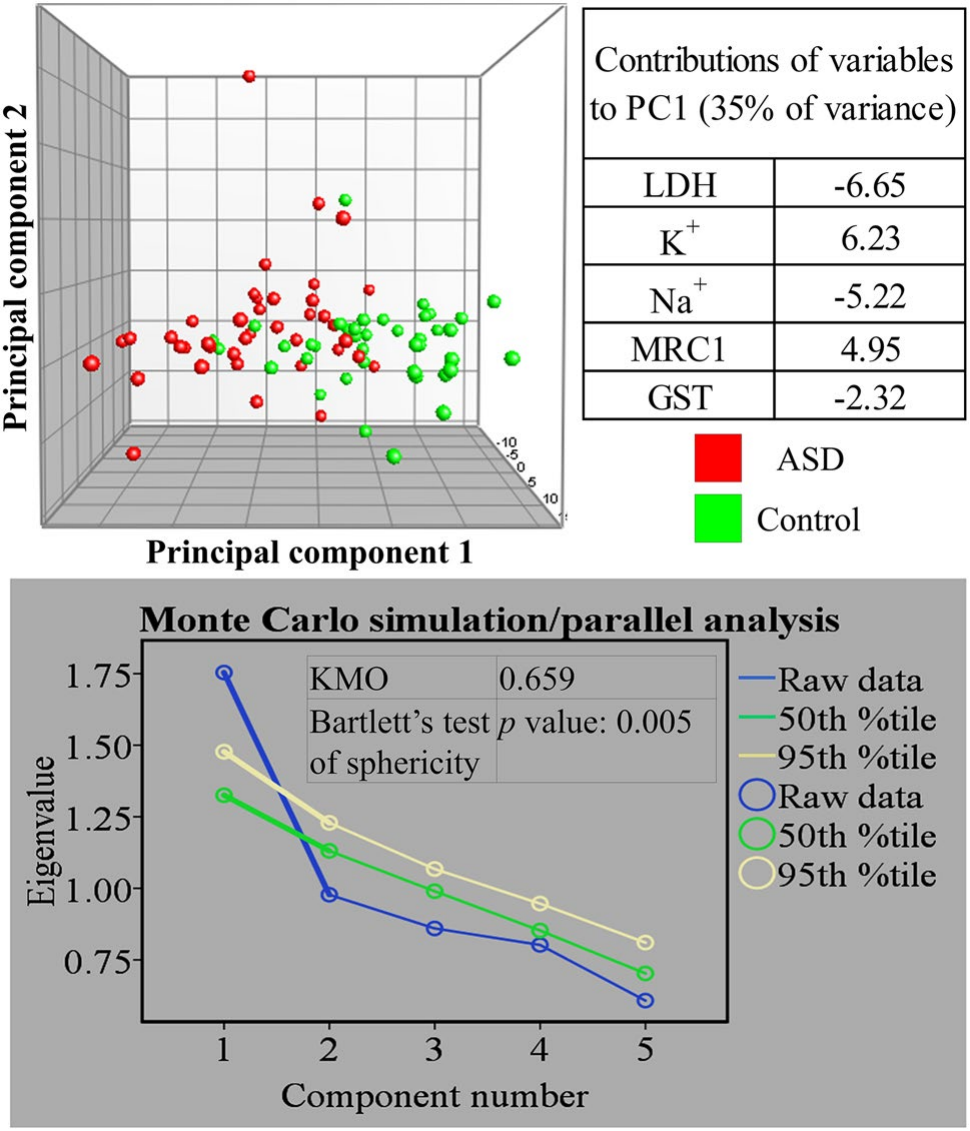
Separation of ASD (n = 40) and age-matched healthy (n = 40) subjects using principal component analysis. The analysis was performed using five variables: potassium (K), sodium (Na), lactate dehydrogenase (LDH), glutathione S-transferase (GST), and mitochondrial respiratory chain complex I (MRC1). Table (top right) shows variable contributions to the first principal component. Graph (bottom) shows the results of Monte Carlo simulation with eigenvalues plotted for raw data and 50th and 95th percentile simulated data. Results of Kaiser–Meyer–Olkin measure of sampling adequacy (KMO) and Bartlett’s test of sphericity are indicated. Figure panels were compiled in Microsoft PowerPoint Slide Presentation Software, Microsoft 365, Microsoft.com. PCA panel (top) was generated in BioNumerics version 6.6, Applied Maths, Austin, Texas, https://www.bionumerics.com. Monte Carlo simulation panel (bottom) was generated in IBM SPSS Statistics for Windows, Version 27.0, IBM Corp., Armonk, New York, https://www.ibm.com.

**Figure 3:**
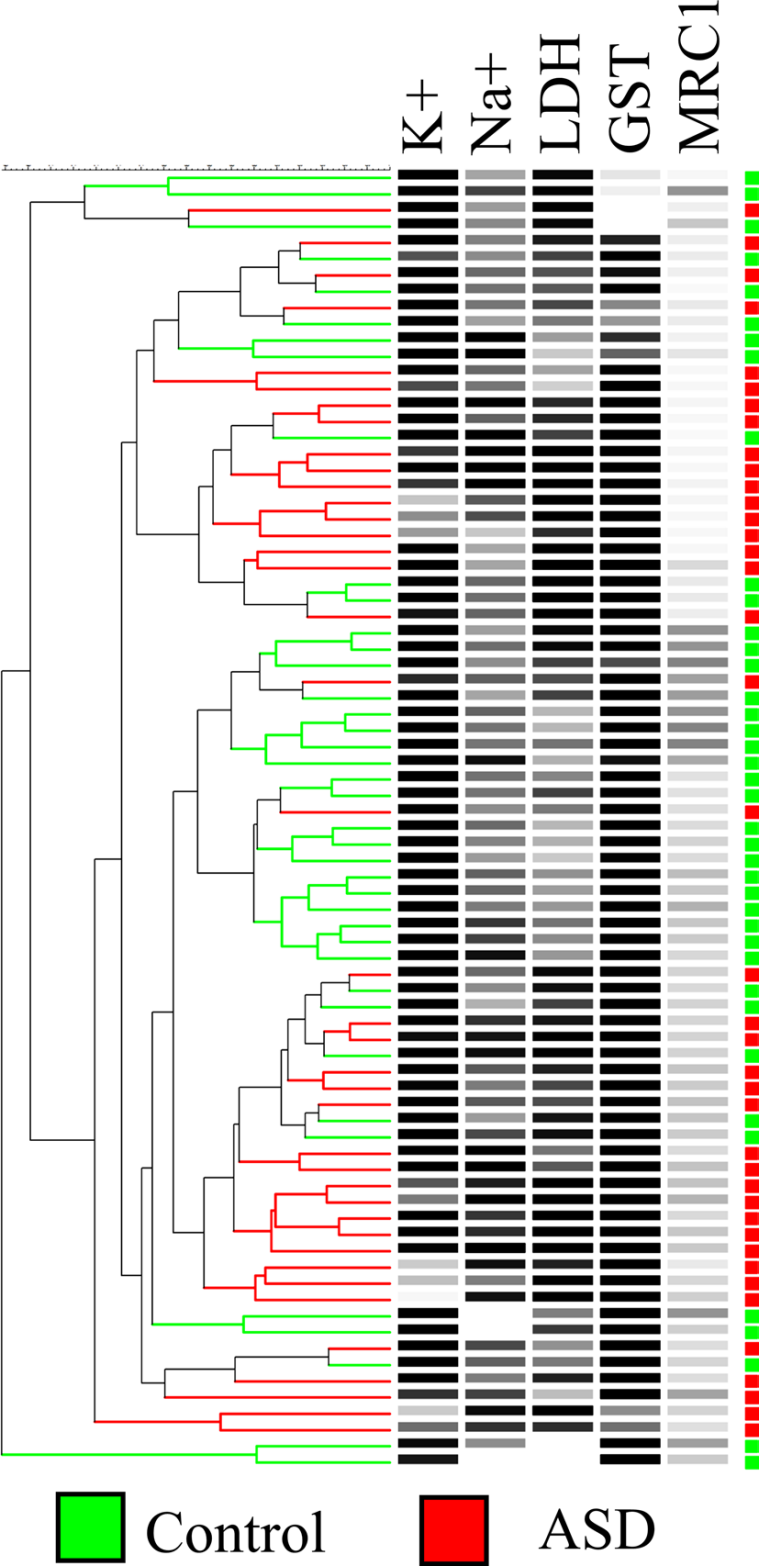
Separation of ASD (n = 40) and age-matched healthy (n = 40) subjects in hierarchical clustering. The dendrogram was constructed using the Unweighted Pair Group Method with Arithmetic Mean (UPGMA) algorithm using a similarity matrix generated using Canberra distances (Eq. 2). Dendrogram branches and corresponding squares (on the far right) are colorized by group (autistic: red, control: green). Black-and-white heat maps represent relative level of the corresponding variables. Figure was generated using BioNumerics version 6.6, Applied Maths, Austin, Texas, https://www.bionumerics.com.

Five plasma variables were tested: K^+^, Na^+^, LDH, GST, and MRC1. Measurements on all five variables significantly differed between ASD patients and age-matched typically developing volunteers as determined by an unpaired student’s t-test (*p* values ranging from 8.7 × 10^7^ to 0.0038) (Fig. 1). The initial evaluation of the utility of the five variables in distinguishing between ASD and control volunteers was accomplished by examining their natural partitioning using PCA and hierarchical clustering. Both methods showed partial separation of ASD patients from control participants. We show using PCA that the separation of the two groups was mostly stretched along PC1. The statistical significance of PC1 was validated using Monte Carlo simulation, but it only explained 35% of the data set variance. A Bartlett’s test of sphericity *p* value of 0.005 indicated that the use of PCA in our data set was appropriate, and a Kaiser–Meyer–Olkin (KMO) of 0.659 was consistent with a barely acceptable sample size. According to our PCA results, LDH and K^+^ were the most important in separating autistic patients from controls as they had the largest contributions to PC1, followed by Na^+^ and MRC1. GST was the least important in this regard (Fig. 2). Hierarchical clustering results showed incomplete separation between ASD and control subjects, with ASD patients predominating two large branches and controls clustering in one central branch of the dendrogram (tree) (Fig. 3).

### Generating a group-membership model and a multivariate biomarker profile using discriminant analysis

We first confirmed the absence of highly correlated variables and the homogeneity of variance across groups using Pearson Correlation Coefficient and Box’s M test, respectively (Table 1a). As explained in the materials and methods section, these criteria are both important whenever the use of DA is considered. We then generated two DA models, one containing all five analytes (all-inclusive model), and another exclusively comprised of the analytes that significantly improved the model (stepwise model). Both models were highly significant, as indicated by their respective Chi-square *p* values (2.66 × 10^–9^ and 2.24 × 10^–9^) and explained more than 45% of data variance as indicated by the corresponding Wilks’ Lambda statistics (Table 1b). The stepwise model contained four analytes: K^+^, GST, MRC1, and LDH, in descending order of their contribution to the model as indicated by their respective standardized canonical discriminant function coefficients. K^+^ had the largest portion of any biomarker’s variance associated with group membership (26.8%), which highlights the importance of this biomarker to the model. Close to 90% of Na^+^ variance did not explain group membership and, therefore, it was not incorporated into the model (Table 1c). The all-inclusive model showed comparable standardized canonical discriminant function coefficients to the stepwise model. Since the Wilks’ Lambda statistic and group means were determined before model construction, these values were identical for both models. Since there were two groups (i.e., ASD and control), a single discriminant function was extracted in each model. The all-inclusive model had slightly higher eigenvalue (0.904) and canonical correlation (0.689) than the stepwise model (eigenvalue: 0.837; canonical correlation: 0.675) (Table 1d). The relatively high eigenvalues of both models indicate that Disc1 explained a large amount of variance in each model; combined with moderate to border-line high canonical correlation, it indicates a discriminant function with fairly high discriminating power. Finally, we evaluated the rate of correct classification (RCC) of ASD and control participants based on our discriminant models. Thirty-one control participants (77.5%) and 34 ASD participants (85%) were correctly classified, amounting to an overall RCC of 81.3% using the stepwise DA model. Using the all-inclusive model, 33 (82.5%) control and 32 (80.0%) ASD participants were correctly classified, also with 81.3% overall RCC (Table 2).

**Table 1:** Discriminant models data.

(a) Data suitability for discriminant analysis						
Pooled within-groups matrices (correlation between predictor variables)						Box’s M test *p* value (cutoff > 0.001)
	K^+^	Na^+^	LDH	MRC1	GST	
K^+^	1	−0.065	−0.215	−0.016	0.12	0.015
Na^+^	−0.065	1	0.136	−0.038	−0.03	
LDH	−0.215	0.136	1	−0.129	−0.103	
MRC1	−0.016	−0.038	−0.129	1	0.049	
GST	0.12	−0.03	−0.103	0.049	1	

(b) Model fitness						
Wilk’s Lambda	*p* value					
0.525/0.545	2.66 × 10^–9^/2.24 × 10^–9^					

(c) Variable contribution to the discriminant model and inter-group difference of means						
	SCDFC	Wilks’ Lambda (equal for both models)	F test *p* value (cutoff < 0.01) (equal for both models)			
K^+^	−0.626/0.661	0.732 (26.8%)	8.66 × 10^–7^			
GST	0.529/−0.547	0.876 (12.4%)	0.0013			
MRC1	−0.392/0.414	0.871 (12.9%)	0.0011			
LDH	0.294/−0.341	0.839 (16.1%)	0.0002			
Na^+^ (excluded stepwise)	0.276/NC	0.898 (10.2%)	0.0038			

(d) Discriminant function discriminatory power						
	Eigenvalue	Canonical correlation				
First discriminant function	0.904/0.837	0.689/0.675				

(a) Test results aiming to evaluate data suitability for discriminant analysis. (b) Model significance and fitness indicators. (c) Indicators of importance of individual variables to the model. SCDFC indicates the contributions made by each variable to the model, Wilks’ Lambda indicates the amount of variance that is unrelated to group membership with the percentage of relevant variance in parentheses, and the F test *p* value indicates the significance of differences between group means for each variable. (d) Canonical discriminant function ability to discriminate between groups. The higher the eigenvalue and canonical correlation, the better the model’s ability to discriminate between groups. Results of the all-inclusive and stepwise models are shown separated by a forward slash in (b), SCDFC column in (c), and (d).

*SCDFC* standardized canonical discriminant function coefficient, *NC* not calculated.

**Table 2:** Rate of correct classification based on discriminant analysis.

Participants’ group	Rate of correct classification	Rate of misclassification	Biomarker merger method
Control	77.5% (31)	22.5% (9)	Discriminant analysis Stepwise (only significant predictor variables)
ASD	85.0% (34)	15.0% (6)	
Overall	81.3% (65)	18.8% (15)	
Control	82.5% (33)	17.5% (7)	Discriminant analysis All-inclusive (all predictor variables)
ASD	80.0% (32)	20.0% (8)	
Overall	81.3% (65)	18.8% (15)	
Control	77.5% (31)	22.5% (9)	Binary logistic regression Stepwise (only significant predictor variables)
ASD	82.5% (33)	17.5% (7)	
Overall	80.0% (66)	20.0% (16)	
Control	80.0% (32)	20.0% (8)	Binary logistic regression All-inclusive (all predictor variables)
ASD	85.0% (34)	15.0% (6)	
Overall	82.5% (66)	17.5% (14)	

Participants [autistic (n = 40) and controls (n = 40)] were classified as either autistic or control based on the discriminant model that had been developed from five plasma biomarkers (potassium, sodium, lactate dehydrogenase, glutathione S-transferase, and mitochondrial respiratory chain complex I). The number of classified subjects is shown in parentheses.

### Generating the binary logistic regression model

A stepwise and all-inclusive BLR models were constructed. The stepwise model was constructed in three steps, all of which were highly significant as indicated by their respective Chi-square *p* values that were lower than 0.05 and Hosmer–Lemeshow *p* values greater than 0.05. The model’s ability to distinguish between ASD and control participants improved at each step as indicated by the progressively increasing Nagelkerke’s pseudo-*R*^*2*^ values (Table 3). The all-inclusive model was also highly significant with a comparable Nagelkerke’s pseudo-*R*^*2*^ value. Considering regression weights, we conclude that MRC1 (highest regression weights) was the most influential in both models, followed by GST and K^+^. Na^+^ and LDH were not incorporated in the stepwise model and were not significant in the all-inclusive model (Table 3). When empirically tested for their ability to correctly classify participants, the all-inclusive model slightly overperformed the stepwise model with overall RCCs of 82.5% and 80.0%, respectively (Table 3).

**Table 3:** Quality assessment of binary logistic regression models.

	Chi-square *p* value	HL test	*R*^*2*^	Variables entered (regression weights/*p* value)
**Stepwise model**				
Step 1	5.19 × 10^–7^	0.833	0.360	K^+^ (0.223/7.4 × 10^–5^)
Step 2	1.85 × 10^–8^	0.215	0.479	K^+^ (0.223/1.4 × 10^–4^) GST (−0.255/0.007)
Step 3	1.82 × 10^–9^	0.840	0.560	MRC1 (0.509/0.014) GST (−0.257/0.007) K^+^ (0.225/3.3 × 10^–4^)
**All-inclusive model (all predictor variables)**				
One step	4.7 × 10^–9^	0.446	0.596	MRC1 (0.419/0.048) GST (−0.222/0.015) K^+^ (0.194/0.003) Na^+^ (−0.010/0.227) LDH (−0.004/0.209)

*R*^*2*^ Nagelkerke’s pseudo-*R*^*2*^ (0–1; 1 corresponds to perfect identification of group membership), *HL* Hosmer–Lemeshow test *p* value (cut-off > 0.05).

### Assessment of the predictive power of potential biomarkers using receiver operating characteristic curves

**Figure 4:**
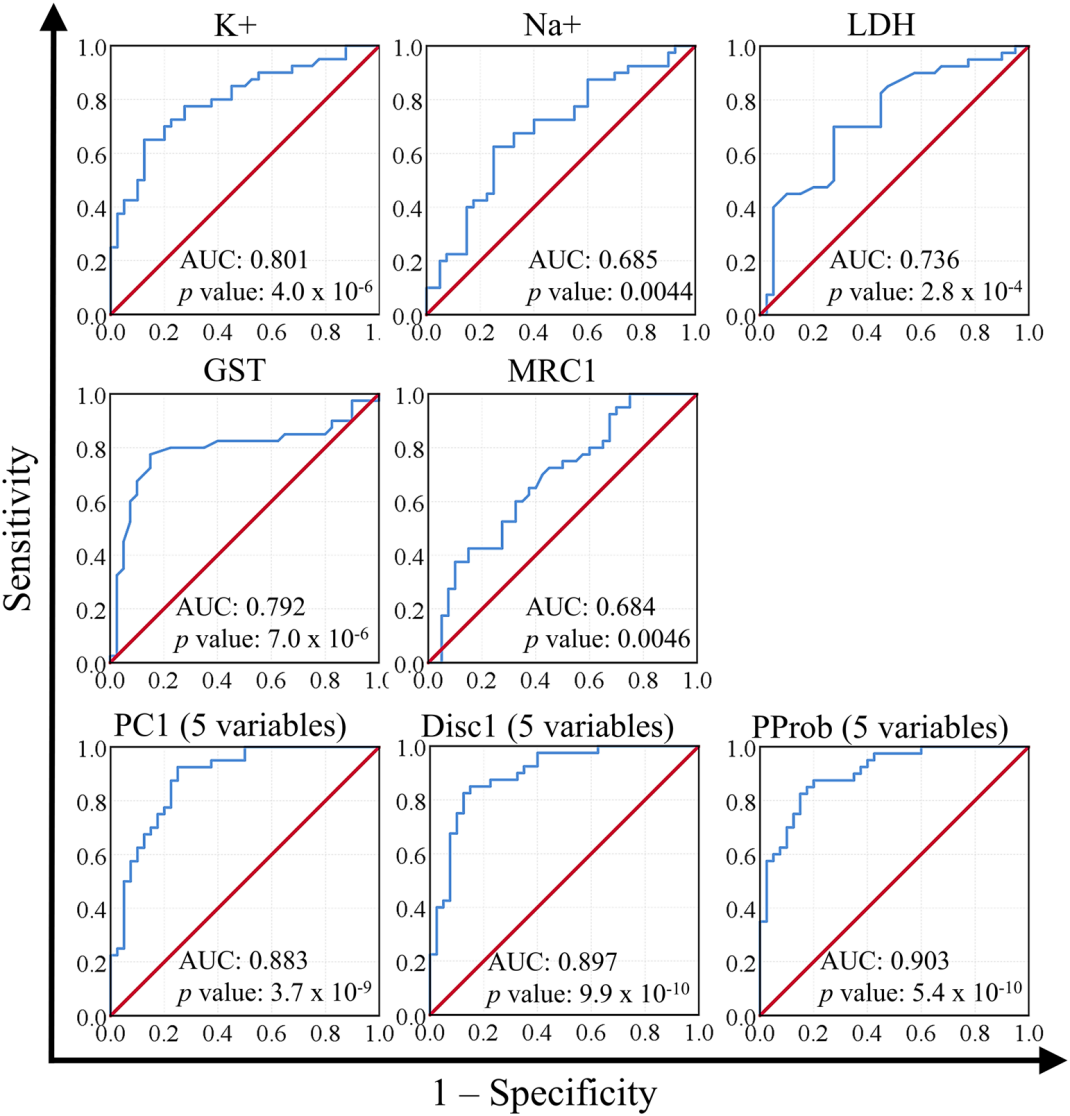
Testing the predictive power of five biomarkers using receiver operating characteristic curve. Areas under the curve (AUC) and *p* values are indicated. Analysis was performed on ASD (n = 40) and healthy (n = 40) volunteers. PC1: first principal component scores computed in principal component analysis. Disc1: first discriminant scores computed in discriminant analysis. *PProb* predicted probability computed by binary logistic regression, *K* plasma potassium, *Na* plasma sodium, *LDH* plasma lactate dehydrogenase, *GST* plasma glutamate S-transferase, *MRC1* mitochondrial respiratory chain complex I activity, *PC1* the first principal component in principal component analysis. Figure was generated using IBM SPSS Statistics for Windows, Version 27.0, IBM Corp., Armonk, New York, https://www.ibm.com.

The next step was to test the predictive power of the five variables individually and in combination, with emphasis on comparing PCA, DA, and BLR. We used the AUC method for this purpose. Our results indicate that K^+^ had the highest AUC (0.801) of any single variable, followed by GST, LDH, Na^+^, and MRC1, respectively. Combining the five variables resulted in higher AUCs than those obtained using single variables. Creating combined variables using PCA resulted in an AUC of 0.883, while using DA and BLR resulted in AUCs of 0.897 and 0.903, respectively (Fig. 4). We also recorded the cutoff value for each variable that corresponded to 80% sensitivity and the corresponding specificity. In line with the AUC results, K^+^ and GST yielded the highest specificity (62.5% and 77.5%, respectively) and PC1 yielded equal specificity to that produced by GST. Both DA and BLR were superior to PCA and comparable to each other (Table 4).

**Table 4:** Sensitivity and specificity as determined by ROC analysis.

Biomarker	Cutoff	Sensitivity (%)	Specificity (%)
K^+^	29.799043	80.0	62.5
Na^+^	148.350000	80.0	40.0
LDH	207.648000	80.0	55.0
GST	8.281250	80.0	77.5
MRC1	3.972000	80.0	40.0
PC1	1.217333	80.0	77.5
Disc1	−0.227751	80.0	87.5
PProb	0.436506	80.0	85.0
Caspase 7	30	27	57
Coenzyme Q10	30	27	57
Melatonin	32	29	61

*PC1* first principal component scores computed in principal component analysis, *Disc1* first discriminant scores computed in discriminant analysis, *PProb* predicted probability computed by binary logistic regression, *K*^*+*^ plasma potassium, *Na*^*+*^ plasma sodium, *LDH* plasma lactate dehydrogenase, *GST* plasma glutamate S-transferase, *MRC1* mitochondrial respiratory chain complex I activity.

## Discussion

In congruence with our previous studies^1,19^ and the studies of other groups^2–4,20–22^, we show that the use of combined biomarkers augments their diagnostic efficacy. In addition, we directly compare the utility of PCA, DA, and BLR in combining potential ASD diagnostic biomarkers. Our results clearly demonstrate that DA and BLR are superior to PCA in discriminating between ASD and control subjects. A pertinent question is whether these results are broadly applicable to other biomarker panels and participant populations. Given that PCA is not computed specifically to maximize the distinction between groups, while DA and BLR are, we predict that DA and BLR will remain superior to PCA regardless of the biomarkers and populations studied. In theory, PCA can be equivalent to DA in differentiating between two groups whenever PC1 is perfectly parallel to Disc1. In such case, the scores of PC1 would likely be as good as Disc1 scores when used to combine biomarkers. The problem is that this is seldom, if ever, the case; there is almost always some degree of diversion between the PC and the line connecting group centroids in DA (i.e., Disc1). In practice, however, combining multiple biomarkers with strong predictive power using PCA has returned perfect AUCs. For example, we have previously reported AUCs equal or close to 1 (i.e., perfect sensitivity and specificity) when using a panel of 7 or 9 biomarkers^1,19^ or the ratios of 5 pairs of biomarkers^19^. In these cases, the predictive power and number of biomarkers seemed to have offset the imperfection of using PCA scores as a means of combining biomarkers. Another advantage of using specifically BLR, but not DA, over PCA is that BLR does not require the absence of collinearity nor does it require homoscedasticity (homogeneity of variance–covariance) across groups^6^. Therefore, BLR is most suitable for data sets that lack these characteristics. We, therefore, encourage the use of DA and BLR in creating multivariate biomarkers in future studies. Although it is likely that our results will be reproducible in the context of other biomarkers and populations, we acknowledge the need for empiric verification of our predictions and precise identification of the limitations and clinical utility of PCA, DA, and BLR. That is particularly true given our sample size, which although is sufficient for PCA and DA by available statistical standards and is comparable to the numbers typically used in phase I trials, it is much smaller than the numbers of participants typically used in phase II and III clinical trials^23^.

There are two approaches when constructing DA and BLR models: one restricts the model to the most useful biomarkers (stepwise), while the other forces all biomarkers into the model (all-inclusive). The stepwise approach can be advantageous as it reduces the number of analytes needed to achieve the distinction between groups and may, therefore, result in cost, labor, and time savings. On the other hand, our data show that incorporating all biomarkers in the model seemed to improve the DA model’s eigenvalue and canonical correlation, and BLR model’s *R*^2^ and RCC. Additional studies are also needed to determine the breadth of applicability of these findings, and whether it is best to use a restrictive approach, such as the stepwise models described here, or a broader range of biomarkers.

In the current study, DA and BLR models differed in their utilization of each of the five analytes tested. The top three most important biomarkers were K^+^, GST, and MRC1, in descending order of importance in DA models and ascending order of importance in BLR models. The outcome of ROC analysis concurred with DA—at least more so than with BLR—since K^+^ and GST yielded the largest AUC and highest specificity among all analytes. In addition, MRC1, which was the most important biomarker in BLR models, along with Na^+^, had the smallest AUCs and lowest specificities. Inquisitively, in the stepwise model of BLR, K^+^ and GST were introduced in the first and second steps, respectively, while MRC1 was not introduced until the third step. Furthermore, K^+^ and GST performed well in larger panels of biomarkers. We have previously reported that GST returned one of the largest AUCs in a nine-biomarker panel (gamma-aminobutyric acid, dopamine, serotonin, GST, vitamin E, mercury, lead, gamma-interferon-inducible protein 16, and oxytocin)^1^ and a twelve-biomarker panel (LDH, glutathione, GST, creatine kinase, coenzyme Q10, caspase 7, and melatonin, lactate, pyruvate, aspartate aminotransferase, alanine aminotransferase, and electron transport chain complex I)^24^. In another study, we showed that K^+^ and GST produced the largest AUCs in another nine-biomarker panel (Na^+^, K^+^, LDH, glutathione, GST, creatine kinase, coenzyme Q10, caspase 7, and melatonin)^19^. Taken together, our data suggest that K^+^ and GST had the highest potential in distinguishing between ASD and control participants, followed by MRC1.

Despite the heterogeneity and the multifactorial nature of ASD and the diverse functions of our biomarkers, our participants showed a homogeneous response across all five biomarkers. This is not unpredictable since these biomarkers are integral to pathways known to be impaired in ASD. Oxidative stress, mitochondrial dysfunction, and channelopathy have all been consistently reported in the local ASD community in Saudi Arabia^18,19,24^. These same dysfunctions have also been linked to ASD in various other geographical locations^10,14,25–28^, implying a global, rather than a local, trend.

The reported potential of blood K^+^ levels in the discrimination between individuals with autism and controls is well supported in the literature and could be related to glutamate excitotoxicity, a recognized pathogenic mechanism implicated in ASD. Several ASD-related SNPs were identified in CNTNAP2, a member of the neurexin family of transmembrane proteins that regulates neuron-astrocyte interactions and K^+^ channel clustering^29,30^. These same variants of CNTNAP2 locus were found to correlate with language impairment, which is a core feature of ASD; reduced number of GABAergic interneurons, which represent an integral part of glutamate excitotoxicity; and abnormal neuronal synchronization^30–32^. A growing body of evidence has linked ion channel dysfunction, including K^+^ channel dysfunction, to vulnerability to autism^14^. K^+^ channel defects may contribute to ASD pathogenesis by altering important brain neural networks. Since a single astrocyte may control the activity of thousands of synapses, defective astrocyte K^+^ ion channels could plausibly contribute to ASD pathogenesis^33,34^. Additionally, treatment with the antipsychotic drug risperidone alleviated excessive grooming and hyperactivity in rodent models of autism, suggesting a potentially useful therapeutic intervention that could improve certain symptoms of autism related disorders and schizophrenia through increasing the number of GABAergic interneurons and potentially restoring the function of CNTNAP2 variants-related defects of K^+^ channels^35,36^. Depletion of intracellular K^+^ can also be related to apoptosis or neuronal death through activation of caspases^37,38^. Multiple studies have shown that altered K^+^ current following glutamate *N*-methyl-d-aspartate (NMDA) receptor activation, a major event in glutamate excitotoxicity, induces apoptotic changes in hippocampal neurons in vitro^39–41^*.*

In addition to K^+^, GST showed a high predictive value, when used as a single biomarker (Fig. 4), compared to the other three variables we have investigated. The central nervous system is particularly sensitive to oxidative stress because of the formation of reactive oxygen species (ROS) concomitant with the alteration of the balance between prooxidant and antioxidant molecules and deregulation of GSH homeostasis^42,43^. The significantly higher utility of GST as an ASD biomarker reported in the present study could be related to epilepsy—a common co-morbidity among ASD patients—and to neurobiological, cognitive, psychological, and social impairments^44^. Recently, resistance to anti-epileptic drugs has been attributed to abnormal GST levels, which is the most important detoxification enzyme known to show altered levels in several neurological disorders^44,45^. GST catalyzes the conjugation of metabolites to GSH, favoring the removal of epoxide metabolites that are generated during the metabolism of antiepileptic drugs^46^. The relevance of MRC1 to ASD is similarly supported by its physiological role as a component of the impaired electron transport chain oxidative phosphorylation bioenergetics known to have profound effects on physiological neurogenesis and on the proper establishment of neuronal function in the brain of ASD patients^24^. Increase of LDH is consistent with altered energy metabolism previously reported in Saudi ASD patients^47^.

Finally, we observed remarkable increases of AUC were observed when combining the five variables (K^+^, Na^+^, LDH, GST and MRC1) using PC1 scores, Disc1 scores, and the PProb from BLR. The increased AUCs could have resulted from combining biologically diverse biomarkers, which might have enabled the proper identification of participants despite ASD heterogeneity.

## Conclusion

Multivariate biomarkers emerge as a potentially powerful tool in ASD diagnostics and beyond. DA and BLR are more suited for creating such multivariate biomarkers, and the latter is more suited for data sets that do not satisfy DA assumptions. Future studies should investigate larger populations and aim to optimize both the mathematical approach and the selection of individual analytes with the ultimate goal of maximizing specificity, sensitivity, and reproducibility across diverse patient populations.

## Materials and methods

### Participants

This work was ethically approved by the ethical committee of King Khalid Hospital, King Saud University (Approval number is 11/2890/IRB). All subjects enrolled in the study had written informed consent provided by their parents and assented to participate if developmentally able. All methods were performed in accordance with the relevant guidelines and regulations. The diagnosis of ASD was ascertained in all ASD participants using the Autism Diagnostic Interview-Revised (ADI-R) and the Autism Diagnostic Observation Schedule (ADOS) and 3DI (Developmental, dimensional diagnostic interview) protocols. The control group was recruited from the well-being pediatric clinic at King Khalid University Hospital. Subjects were excluded from the investigation if they had dysmorphic features, or diagnosis of fragile X or other severe neurological (e.g., seizures), psychiatric (e.g., bipolar disorder) or known medical conditions. All participants were screened through parent conversation for current and earlier physical illness. Children with known pulmonary, cardiovascular, endocrine, liver, kidney, or other health problems were excluded from the study. All patients and controls were receiving average local diet and were not on any nutrient-restrictive diet. Forty male mild-moderate ASD patients and 40 typically developing participants were included in the study (Table 5). Data for 13 ASD patients and 24 control participants have been included in a previous study investigating nine biomarkers, including four of the five biomarkers investigated in this study (K^+^, Na^+^, LDH, and GST)^19^. Using fewer variables in this study enabled the inclusion of a larger number of participants than what was possible in the previous study. We also included MRC1 in the current study, which was not included in previous work.

**Table 5:** Demographic data of autistic and control participants.

	ASD (N = 40)	Control (N = 40)
Age/years	7.5 ± 4.28	7.6 ± 3.96
Males	40	40
Females	0	0
Born by caesarian section	60%	30%
Affected individual/family	1	0
Paternal age/year	37.06 ± 4.23	34 ± 4.82
Maternal age/year	28.56 ± 4.27	27.96 ± 4.03
Prematurity	0	0

### Specimen collection

Whole blood samples were collected by venipuncture after overnight fasting. Each 10 ml sample was collected in heparin tubes. Plasma was purified by centrifugation promptly after sample collection and was store at − 80 °C until used for analysis.

### Biochemical assays

Plasma levels of K^+^, Na^+^, LDH, GST, and MRC1 were measured according to the protocol previously published by Khemakhem et al.^24^. K^+^, Na^+^, LDH were measured using diagnostic kits, products of United Diagnostics Industry (UDI), Dammam, and KSA. GST was measured using spectrophotometer at 340 nm, and activity was indicating in μmol/mL/min^23^. Positive and negative controls were measured to check the validity of the measurement, and to determine the detection limits. MRC1 was measured using ELISA kit, product of MyBiosource USA. This kit is suitable to assay the levels of ETCComplex I in undiluted human plasma samples using a quantitative sandwich ELISA technique. Detection limit of this kit is 3.12–100 ng/ml.

### Principal component analysis

PCA was performed using either BioNumerics version 6.6 (Applied Maths, Austin, Texas) or IBM SPSS version 24 (IBM Corporation, Armonk, NY) as previously described^1,48^. Briefly, PCA was performed on covariance matrices and data were normalized by subtracting the mean and dividing by the variance. Normalization was performed to minimize biased contributions of variables to PCs that may result due to unequal scale across variables. In other words, normalization was performed to eliminate the dominance of variables expressed in large numerical values and the underrepresentation of variables expressed in small numerical values. Bartlett’s test of sphericity provided a *p* value that represents the likelihood that a data set has no correlated variables. In the absence of correlated variables, PCA generates as many PCs as variables with each representing one variable, which makes the use of PCA in such data sets useless. Therefore, a *p* value < 0.05 is required for PCA to be useful^49^. KMO measure of sampling adequacy was used to evaluate the adequacy of sample size for PCA to be meaningful^50,51^.The significance of principal components was determined using Monte Carlo simulation—also known as parallel Analysis—using Brian O’Connor’s syntax for SPSS^52^. Bartlett’s test of sphericity, KMO, and Monte Carlo simulation were performed using IBM SPSS version 24.

### Discriminant analysis

A few verification tests were performed to confirm the suitability of the data for DA. Predictor variables should not be highly correlated^53^, which was determined by inspecting a Pearson Correlation matrix that can be found in SPSS DA analysis output under “Pooled Within-Groups Matrices”. Correlations with *r* <|0.5| were considered acceptable in the current study. Variance–covariance homogeneity, which is one of the assumptions of DA, was tested using Box’s M test. The null hypothesis of Box’s M states that dependent variables covariance matrices are equal across groups, which needs to be retained to satisfy the assumption of covariance matrices homogeneity^54^. Box’s M null hypothesis is rejected at a *p* value > 0.001^55^. Our sample size is 80 participants, 40 per group. Sample size requirement in DA and similar techniques is not well defined in the literature. Based on currently available data, it has been suggested that the size of the smallest group in a data set should outnumber the independent variables by at least three-fold^56^. Since we have five independent variables, our sample size well exceeds this standard. The overall significance of the model was evaluated using the Wilks’ Lambda statistic, which corresponds to the proportion of discriminant function variance that cannot be explained by differences in group membership (i.e., variance in a single discriminant or a set of discriminants that is nonpredictive of group membership). Therefore, Wilks’ Lambda is a “badness-of-fit” measure with lower values indicative of a better discriminant model. The values of the Wilks’ Lambda statistic may range from 0 to 1, with 0 indicating perfect group discrimination and 1 indicating lack of any discrimination. A chi-square statistic is used to test the null hypothesis stating that the discriminant model is as good as random chance alone, which is rejected at *p* values < 0.05^6^. We have also evaluated the efficacy of discriminant functions and the relative importance of each of the five biomarkers for group discrimination. Indicators of efficacy of discriminant functions include eigenvalues and canonical correlations. The higher the eigenvalues, the higher the amount of variance a discriminant function explains. Canonical correlation is the function’s correlation with the groups, with more efficacious functions having higher correlations. The importance of individual biomarkers to the model was evaluated in two ways. One way was to evaluate the ability of each biomarker to discriminate between groups without controlling for its correlation with other biomarkers. To accomplish this, two values were considered. The significance of differences in group means on each variable was tested using an F-test with a Bonferroni-corrected *p* value of 0.01 (0.05/number of variables)^6^ .The other value we used to evaluate the importance of individual biomarkers was the Wilks’ Lambda statistic, which showed how much of the biomarkers variance was not explained by inter-group differences; the closer this value is to zero, the better the discriminatory power of the corresponding biomarker in isolation (as opposed to as part of a model^6^.The other way individual biomarkers were evaluated was by looking at their scalers (i.e., standardized canonical discriminant function coefficients), which directly measures the contribution of biomarkers to the discriminant model. The model is further validated by calculating the rate of correctly classifying participants into their respective groups based on the model, or RCC. For the purposes of RCC calculations, the discriminant model was recalculated for each classification step (i.e., for each participant), with the participant being classified left out of the model. RCC was compared when using stepwise DA versus DA performed with all independent variables incorporated into the model. DA and associated tests were performed using IBM SPSS version 24.

### Binary logistic regression

BLR uses data from one or more predictor variables (e.g., biomarkers) to predict the odds of a binary dependent variable (e.g., odds of being diagnosed with ASD or being free of such diagnosis). The odds are calculated using Eq. (2). Since the odds themselves rarely form a linear relationship with the dependent variable, the predictive model is built around the natural log of odds (*L*_*i*_). *L*_*i*_ is computed by selecting a regression coefficient for each predictor variable aiming to maximize the goodness of fit of the model (Eq. 3). Regression coefficients of each predictor variable represent the average change in this variable with each unit change in the dependent variable while accounting for the effects of other independent variables. The odds and probability of falling into either group (i.e., ASD or control) can then be calculated from *L*_*i*_ using Eqs. (4) and (5), respectively^6^. The significance of the model is evaluated using a Chi-square test that tests whether incorporating predictor variables into the model caused significant improvement over the null model (i.e., a model with no predictor variables). Significant models will have *p* values < 0.05. Further testing is done to evaluate the quality of improvement afforded by the model over the null model, for which we used the Hosmer–Lemeshow test and the Nagelkerke’s pseudo-*R*^2^. The null hypothesis of Hosmer–Lemeshow test is that the model predicts group membership with perfect accuracy, which is retained with *p* values > 0.05^57^. Nagelkerke’s pseudo-*R*^*2*^ takes values between zero and one. The closer Nagelkerke’s pseudo-*R*^*2*^ to one, the higher the model’s quality^58^. Similar to DA, BLR can incorporate all variables or sequentially add variables starting with the variable that introduces the most significant model improvement and ending when incorporating more variables into the model results in no significant improvement. The RCC was compared using both approaches. BLR was performed using IBM SPSS version 26.

2 $$Odds\,  of\,  falling\,  in\,  the\,  autistic\,  group=\frac{P}{1-P},$$

where *P* is the  probability of falling in the ASD group and $1-P$ is the probability of falling in the control group.

3 $${\text{L}}_{\text{i}} = {\text{ln}}\left(\frac{P}{1-P}\right) = {  {\text{B}}}_{0} { + }{\text{B}}_{1}{{\text{X}}}_{1 } {+}{\text{ B}}_{2}{{\text{X}}}_{2}{ +\cdots}{\text{ B}}_{\text{i}}{{\text{X}}}_{\text{i}}{\cdots + }{{\text{B}}_{\text{n}}}\,{{\text{X}}_{\text{n}}},$$

where *L*_*i*_ is the natural log of odds, *ln* is the natural log, *P* is the probability of falling in the ASD group, $1-P$ is the probability of falling in the control group, *B*_*0*_ is the intercept, *B*_*i*_ is the *i*th logistic regression coefficient, and *X*_*i*_ is the *i*th predictor variable.

4 $${\text{Odds}} \, {=}\, {\text{e}}^{{\text{L}}_{\text{i}}} =  {\text{e}}^{{\text{B}}_{0}{ + }{\text{B}}_{1}{{\text{X}}}_{1 }{+}{\text{ B}}_{2}{{\text{X}}}_{2}{ + \cdots + }{\text{B}}_{\text{n}}\,{{\text{X}}}_{\text{n}}},$$

where *L*_*i*_ is the natural log of odds and *e* is the base of the natural log and is approximately equal to 2.71828.

5 $${\text{Pi}}{= }\frac{{\text{e}}^{\text{Li}}}{{1 + }{\text{e}}^{\text{Li}}} ,$$

where *P*_*i*_ is the probability of falling in the ASD group for the *i*th participant, *L*_*i*_ is the Logit statistic, and *e* is the base of the natural log and is approximately equal to 2.71828.

### Hierarchical clustering

Hierarchical clustering aims to organize a data set in such a way that similar data points are grouped together in clusters. These clusters are displayed in the form of a tree or a dendrogram. The first step in hierarchical clustering is to calculate a similarity matrix composed of all possible pairwise similarities in the data set. In the current study, we used Canberra distances (Eq. 6) to calculate similarity matrices. Dendrograms are then constructed from these similarity matrices in one of two ways. One way uses divisive (top-down) algorithms that start with all data points in one group that are gradually divided into branches. The other way uses agglomerative (bottom-up) algorithms that start with individual data points that are gradually linked into clusters^59^ In the current study, we used the *U*nweighted *P*air *G*roup *M*ethod with *A*rithmetic Mean (UPGMA) algorithm to construct our dendrograms since it gave us the most easily discernable segregation between ASD and control participants (data not shown). UPGMA is an agglomerative algorithm that initially links the most similar pair of data points to form the first cluster. It then treats the newly formed cluster as an individual, recalculates the similarity matrix using the first cluster as an individual data point, and links the most similar pair forming a second cluster. This process is repeated until all data points are joined into one dendrogram^60^. Hierarchical clustering was performed using BioNumerics versions 6.6.

6 $$D= \frac{1}{n}{\sum}_{i=1}^{n}\frac{\left|Xi-Yi\right|}{\left|Xi + Yi\right|},$$

where *D* is the Canberra distance, *n* is the number of data points, and *X* and *Y* are the data points being compared in any given pairwise comparison.

### Receiver operating characteristic curve

The predictive power of biomarkers was evaluated by calculating AUC. AUC calculation was done in IBM SPSS version 26 as previously described^1^. Briefly, an AUC of 1 corresponds to 100% sensitivity and 100% specificity, while an AUC of 0.5 indicative of the complete lack of predictive power^61^. Biomarker profiles used in ROC analyses were constructed by performing PCA, DA, or BLR and substituting the observed data by the scores of the principal component responsible for most of the segregation between the ASD and control groups, the scores of Disc1, or PProb, respectively. To select the principal component responsible for most group separation, participants were plotted on the coordinates of the first 3 components (PC1, PC2, and PC3). The resulting three-dimensional plots were visually inspected to identify the PC on which most of the group separation occurred. Visual inspection was augmented by the ability to rotate these plots in BioNumerics. All variables were incorporated into PCA, DA, and BLR models for the purposes of this analysis.

### Other statistical analysis

Two-tailed student’s t-test was performed in Microsoft Excel (Microsoft Technology Company, Redmond, Washington).
