# Author Correction: Discriminant analysis and binary logistic regression enable more accurate prediction of autism spectrum disorder than principal component analysis

**DOI:** 10.1038/s41598-022-23620-z

**Published:** 2022-11-09

**Authors:** Wail M. Hassan, Abeer Al-Dbass, Laila Al-Ayadhi, Ramesa Shafi Bhat, Afaf El-Ansary

**Affiliations:** 1 Department of Biomedical Sciences, University of Missouri-Kansas City School of Medicine, Kansas City, MO USA; 2 Biochemistry Department, College of Sciences, King Saud University, Riyadh, Saudi Arabia; 3 Department of Physiology, Faculty of Medicine, King Saud University, Riyadh, Saudi Arabia; 4 Autism Research and Treatment Center, Riyadh, Saudi Arabia; 5 Central Research Laboratory, Female Centre for Scientific and Medical Studies, King Saud University, Riyadh, Saudi Arabia

Correction to: *Scientific Reports* 10.1038/s41598-022-07829-6, published online 08 March 2022

The original version of this Article contained an error in Figure 4, where an incorrect figure was displayed. The original Figure 4 and accompanying legend appear below.

**Figure 4:**
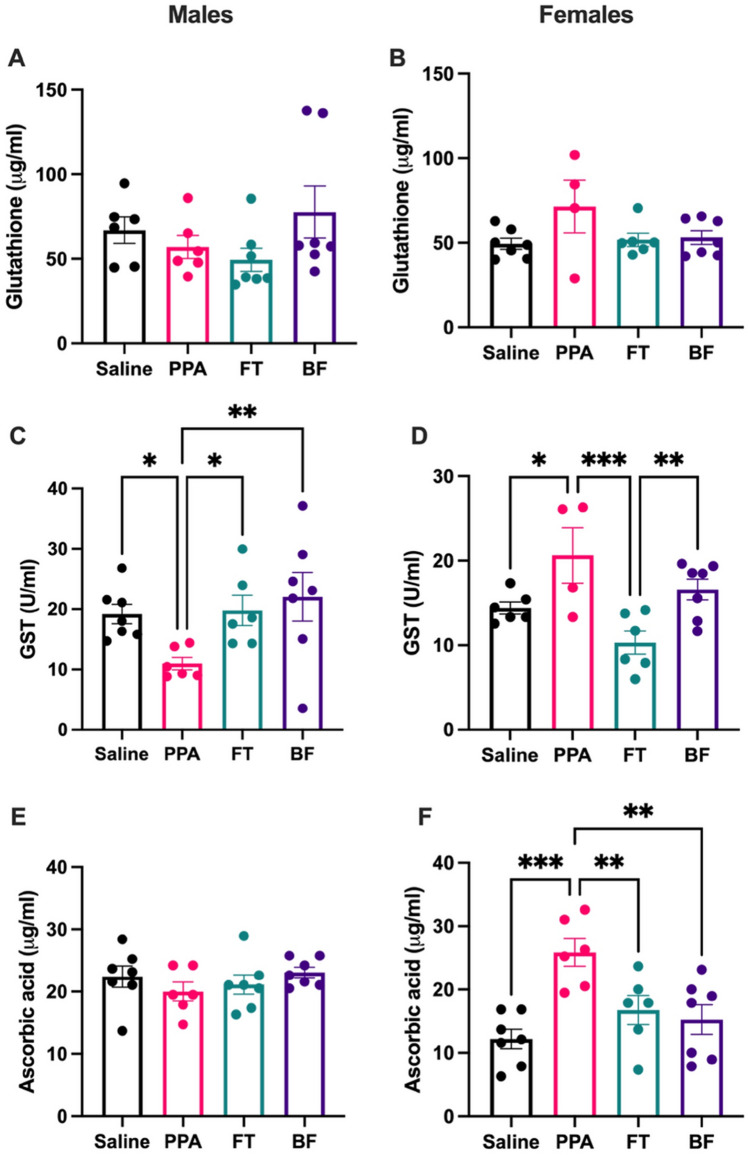
Testing the predictive power of five biomarkers using receiver operating characteristic curve. Areas under the curve (AUC) and *p* values are indicated. Analysis was performed on ASD (n = 40) and healthy (n = 40) volunteers. PC1: first principal component scores computed in principal component analysis. Disc1: first discriminant scores computed in discriminant analysis. *PProb* predicted probability computed by binary logistic regression, *K* plasma potassium, *Na* plasma sodium, *LDH* plasma lactate dehydrogenase, *GST* plasma glutamate S-transferase, *MRC1* mitochondrial respiratory chain complex I activity, *PC1* the first principal component in principal component analysis. Figure was generated using IBM SPSS Statistics for Windows, Version 27.0, IBM Corp., Armonk, New York, https://www.ibm.com.

The original Article has been corrected.

